# Nanomedicines for Efficient Diagnosis and Treatment of Pediatric Respiratory Diseases: Prospects and Challenges

**DOI:** 10.1049/nbt2/2692282

**Published:** 2026-08-12

**Authors:** Yan Shi, Hui Ma, Han Wenchao, Dan Li

**Affiliations:** ^1^ Department of Pediatrics, Qingdao Hospital, University of Health and Rehabilitation Sciences (Qingdao Municipal Hospital), Qingdao 266071, China; ^2^ Department of Neonatal Intensive Care Unit (NICU), Qingdao Women and Children’s Hospital, Qingdao 266071, China, qdu.edu.cn

**Keywords:** clinical translation, nanocarriers, nanomedicine, pediatric respiratory diseases, preclinical studies, pulmonary drug delivery, theranostics

## Abstract

Pediatric respiratory diseases are a major burden of global disease because they are very common and their pathophysiological mechanisms are complex. Conventional therapies have suboptimal pulmonary deposition, systemic toxicity, and poor patient compliance in children. Nanomedicine based interventions provide targeted pulmonary delivery, promote increased bioavailability, controlled drug release and reduce toxicity. Pulmonary deposition, epithelial uptake and intracellular trafficking are regulated by the physicochemical properties and the functionalization of nanoparticles. Preclinical studies in juvenile animal models show superior pharmacokinetic (PK) properties, preclinical therapeutic efficacy and preliminary clinical studies in pediatric patients show favorable safety and tolerability characteristics. There are still challenges associated with translation, such as immunotoxicity, regulatory issues, and optimizing doses in children. New strategies that combine AI‐assisted design and synthesis of nanoparticles, nanotheranostics, and biodegradable 2D nanomaterials have potential to address these challenges. In this review, recent advances in pediatric nanomedicine for respiratory diseases are discussed with special focus on mechanistic understanding, preclinical evidence, translation to the clinics and future directions of the therapeutic platforms.

## 1. Introduction

**Figure 1:**
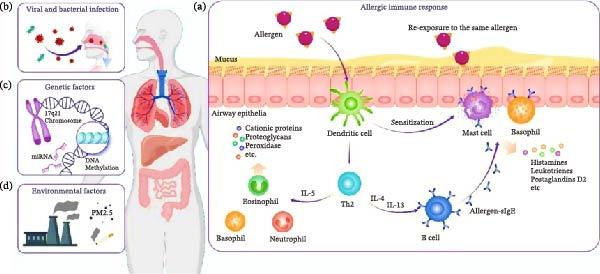
Schematic overview of the multifactorial pathophysiology of airway inflammatory diseases. (a) Allergic immune responses mediated; (b) respiratory viral and bacterial infections; (c) genetic predispositions including polymorphisms affecting airway structure, immune signaling, and cytokine regulation; (d) environmental exposures like pollutants, allergens, and tobacco smoke. Adapted from Ref. [3].

Respiratory tract infections (RTIs) are a health burden of pediatric patients, mostly caused by viral and bacterial infections. Majority of the infections are caused by viruses, such as respiratory syncytial virus (RSV), rhinovirus, adenovirus, influenza viruses, parainfluenza viruses, and human metapneumovirus, but bacteria, such as *Streptococcus pneumoniae*, group A *β*‐hemolytic streptococci, and Mycop. The coinfection of viral and bacterial agents can also increase the severity of the clinical case and make it difficult to manage. The partial advantage of maternal antibodies to neonates is that infants and toddlers are more vulnerable because of the immature state of adaptive immunity, and school‐aged children are more susceptible to bacterial infections as adaptive immunity matures [1, 2]. Younger children are likely to experience the rapid spread of pathogens and the development of secondary complications because of anatomical factors such as narrow airways, shorter respiratory tracts, and the horizontal arrangement of the Eustachian tubes. Comorbidities (asthma and cystic fibrosis [CF]), nutritional status, and environmental exposures (passive smoking and childcare attendance) also affect immune competence. The incidence of viral RTIs tends to rise in the winter and early spring, while that of *Mycoplasma pneumoniae* infections is elevated during autumn and early winter. All of these etiological, anatomical, immunological, and environmental factors, acting together, define the epidemiology and clinical picture of respiratory infections in pediatrics (Figure 1), and in this context, it is crucial to be able to make a correct diagnosis and to have a specific therapeutic approach [2, 4–6].

Traditional management of respiratory infections in children, such as the use of antibiotics, antivirals, and corticosteroids, has major limitations that inhibit their effectiveness and safety. Most of them are viral and are a consequence of RSV and rhinoviruses, but there are no effective antiviral drugs to treat them, and they are not approved to be used in children, leading to less than ideal treatment outcomes. The excessive prescription of antibiotics in case of viral infection also provides an additional contribution to the development of resistant strains, making it harder to control secondary bacterial infection and causing hospitalization [7–9]. There are especially significant differences in the pharmacokinetics (PKs) and the pharmacodynamics of children, so the corticosteroid therapy, in particular, may cause growth retardation, decreased immunity, and infection vulnerability. Also rapid mucociliary clearance decreases deposition and retention of inhaled drugs further weakening the action of traditional therapies [10–12].

Nanotechnology has the potential to maximize pulmonary deposition by regulating particle size and surface charge as well as functionalization to enhance mucosal penetration and retention at site of infection [13–15]. They have a Controlled release which allows for a more steady dose of the medication and reduces the number of doses and side effects to other body systems and increases compliance rates, particularly in chronic respiratory diseases. Nanoparticles can cross biological barriers such as mucus layers and tight junctions of epithelia and enhance the effectiveness of drugs and can be used to deliver drugs to areas that were previously inaccessible [16, 17]. Therapeutic agents can be added to a diagnostic marker, thus making it possible to monitor changes in disease progression and create individual treatment plans. The use of nanomedicines as a means to overcome some of the limitations encountered in the traditional therapeutic approaches is the ability to deliver the drug to the site of infection, thus reducing systemic exposure and toxicity and delivering a more favorable therapeutic profile in children [18–21].

On the other hand, children with respiratory diseases require a delivery system that considers anatomical and physiological uniqueness of the developing lung. Children have narrow airways, reshape their alveoli later into life, have higher respiratory rates, and as infants tend to inhale through the nose. These considerations adjust the airflow, aerosol deposition in the area, and the particle retention. At the same time, the immune system of children continues to develop, and it is this factor that may influence the host reactions to inhaled therapeutics and nanocarriers. In consequence, there exists no scaled‐down adult treatment of instillation of drugs into the lungs of children [22].

Such developmental variations bring about a powerful argument in favor of the pediatrics‐oriented nanomedicine. Traditional inhaled treatments typically exhibit low efficacy in children as very minimal proportions of the given dose get to the peripheral lung, whereas large proportions get wasted in the device, interface, and upper respiratory system. Pediatric therapy is often based on off‐label extrapolation of adult dosing schedule in current practice that creates doubt in efficacy and safety. This is especially critical with nanoparticle‐based systems because the biodistribution and pulmonary retention of nanoparticles, as well as systemic exposure, might vary significantly between pediatric and adult profiles, in particular among neonates and young children. Moreover, age‐related changes in breathing, airway structure, and mask or interface efficiency provide a distinct device‐patient mismatch that further diminishes delivery efficiency and increases dose control [22, 23].

Collectively, these reasons justify why pediatric respiratory nanomedicine needs to be a separate discipline and not a derivative of general pulmonary nanomedicine. Children should have formulations tailored to age‐related factors, such as deposition behavior, PKs, safety, immune compatibility, and device performance. Despite the potential to enhance pulmonary targeting using nanomedicine, decrease off‐target exposure and increase the effect of the therapy, the existing evidence in children is scarce. Such a gap underscores the need to enhance the concept of pediatric‐specific design principles and translational research in inhaled nanomedicines [24].

The use of nanomedicine in respiratory care among the pediatric population can be used not only in the treatment but also in the diagnosis process, particularly in cases where noninvasive sampling is a challenge. Poor sputum production, low volume of samples, invasive collections, and delayed samples are common limitations in respiratory diagnostics in children. They can be overcome with nanomedicine‐based platforms that enhance sensitivity and allow nonsputum or noninvasive testing with low volumes. Examples of applications relevant to pediatrics are serum nanopore assays of tuberculosis, nanoparticle‐enhanced extracellular vesicle blood assays, colloidal gold paper strips to determine rapid severity, and emergent breath or exhaled breath condensate sensors to perform noninvasive screening [25–27].

Blood‐based tuberculosis assays are among the most potent pediatric models. A nanopore serum assay, which transduced *Mycobacterium tuberculosis* antigens into amplified nucleic acid signals, displayed 94.4% sensitivity in confirmed pediatric tuberculosis, versus 44.4% sensitivity in Xpert MTB/RIF and 72.2% sensitivity in culture in 75 children. A nanoparticle‐enhanced extracellular vesicle immunoassay detected 58 of 78 cases of pediatric tuberculosis (equivalent to 74% sensitivity) and 48 of 66 cases false‐negative with conventional microbiological testing in 147 hospitalized immunosuppressed children with HIV on treatment. These studies reveal that nanotechnology has the ability to enhance the diagnosis of pediatrics by lowering testing that involves the use of sputum [25, 26].

Rapid assays using nanomaterials are also promising in the stratification of pediatric severe disease detection. A colloidal gold paper strip designed to detect interleukin‐6 in pediatric serum was used to differentiate severe and mild influenza, and the detection limit was reduced to ~76.85 pg/mL, and results were produced in ~20 min. This assay has been combined with C‐reactive protein to give a sensitivity, specificity, and AUC of 85.7, 95.5, and 0.911, respectively [28]. Condensate platforms of breath and exhaled breath provide another noninvasive direction, yet they are not as validated in pediatrics [27, 29, 30]. These combined results confirm the diagnostic utility of nanomedicine in pediatric respiratory disease as well as indicate that the pediatric diagnostic evidence base is a narrower range of literature than the therapeutic literature.

## 2. Anatomical and Physiological Differences in Pediatric and Adult Respiratory Systems

### 2.1. Pediatric Airway and Lung Development

The cardinal clinical principle is that the lung of a child is not a smaller version of the lung of an adult but an organ that exists in a state of structural and functional flux. Lung development involves steps, namely pseudoglandular, canalicular, saccular, and alveolar stages, with most of the alveolarization taking place after birth and persisting in teenage years [31]. The geometry of the upper airway is also different in infants, with a different angle of the epiglottis and glottis‐to‐cricoid ratio, which locate the narrowest functional range at the cricoid level, as opposed to the adult vocal cords [32]. This geometrical variation produces different airflow resistance and turbulence outlines, which have direct effect on the first direction inhaled nanoparticles will take. In addition, nanocarriers are distributed to the rapidly growing surface area of alveoli in children, the surface area being constructed of different cells, which may have an impact on the biological fate of the nanocarrier’s content [33, 34].

### 2.2. Aerosol Deposition and Clearance

Physiological parameters such as high resting respiratory rate, low functional residual capacity, and preferential nasal breathing in the first 6 months of life undergo significant changes in nanoparticle deposition patterns [35]. Children have smaller tidal volumes resulting in greater local tissue concentrations of the same mass inhaled than in adults because the same mass is deposited on a much smaller pulmonary surface [34]. Simulation studies indicate that child deposition is scaled to Stokes number, with the optimal deposition windows of aerosols being dependent on age and on the delivery device [24]. At smaller sizes (below 100 nm) of the particles, Brownian diffusion predominates, resulting in an extremely high deposition (>95) in the distal airways of modeled pediatric lungs [36]. Compared to adult mucus, which has been shown to exhibit shear‐thinning and elastic behavior, there is little quantitative data comparing the viscosity of pediatric and adult airway mucus and pH. Nevertheless, it is known that the pediatric upper airway has a unique mucosal microclimate with the potential to affect the mucociliary clearance and residence time of mucoadhesive nanocarriers [37–39].

### 2.3. Barrier and Immune Maturation

The risk of translocation of nanoparticles to the system is largely determined by the maturation of the respiratory epithelial barrier and the pulmonary immune system. The air‐blood barrier of immature neonatal lungs is more permeable, and the small nanoparticles (e.g., gold) are much more readily translocated systemically in immature patients than in adults. This translocation seems less size‐selective in the neonates, so there seems to be more dependence on paracellular transport pathways and less on the transcellular pathways prevalent in adult epithelia [36]. Children have a more pronounced early innate mucosal cytokine response, being higher in terms of IFN‐*α*2, IL‐8, and IP‐10 in airway secretions compared to adults. Alveolar macrophages are the main clearance route of deep‐lung nanoparticles, although the functional capacity and inflammatory responsiveness of this clearance route are immature in early childhood and thus can result in slower clearance of nonbiodegradable nanocarriers and an increased risk of chronic inflammation [38, 40].

### 2.4. Implications for Nanocarrier Design

These physiological differences necessitate a shift toward adult‐oriented, down‐scaling in childhood nanomedicine. The design of nanocarrier should be oriented to the following range of Stokes number (typically Stk = 0.030.06) that is optimum in the case of regional targeting of a child with distinct inhalation flow and airway structure [24, 32]. Since smaller nanocarriers are more likely to cause systemic translocation in neonates, it is advised that nanocarriers with surface modifications (e.g., modestly increased aerodynamic diameters or surface‐based stealth coatings) or size distributions (e.g., modestly increased aerodynamic diameters or stealth coatings) be developed into nanocarriers that diminish unintended paracellular passage into the bloodstream [34, 35]. Design approaches should focus on favoring mucopenetrating chemistries to access the heterogeneous microspaces of the pediatric airway and avoid capture by the active mucosal immune response [37, 39]. Finally, primary pediatric air‐liquid interface (ALI) models are needed to test such designs to ensure that safety profiles and efficacy profiles reflect the biological peculiarities of developing lung [40].

## 3. Physicochemical Considerations of Nanocarriers for Pulmonary Delivery

### 3.1. Fabrication Methods and Translational Relevance

Techniques of manufacture can strongly influence particle size, aerosol dynamics, reproduction, and translational viability of inhaled nanomedicines (Table 1). Recent work has indicated a specific shift in noncomplex nanoparticle preparation to process‐directed engineering with the objectives of device compatibility, manufacturing control, and clinically relevant aerosol behavior. Of special interest to pulmonary delivery is the pediatrics where the particle aspects can be easily manipulated to alter the lung deposition and dose delivered.

**Table 1:** Effect of fabrication methods on physicochemical considerations of nanocarriers for pulmonary delivery.

Method	Particle size control	Reproducibility and scale	Aerosol performance	Encapsulation efficiency/stability	Translational potential	Reference
Nanoprecipitation	Produces nanoscale suspensions that define starting NP size	Insufficient evidence	Initial NP size influences final FPF after SFD	Insufficient evidence	Used as a precursor platform for inhalable systems	[41]
Spray drying/nano spray drying	~300 nm to 5 µm with narrow distributions	Tunable via process parameters, improved reproducibility	High emitted dose and FPF, excipients improve dispersion	High yields up to ~90%, preserves structure and release	Demonstrated DPI compatibility and scalability	[42, 43]
Spray‐freeze drying	Porous particles ~5 to 10 µm, FPF 19% to 79%	Strong dependence on excipients	High dispersibility with optimized formulations	Not directly quantified	Converts nanosuspensions into inhalable powders	[44, 45]
Microfluidic aerosolization	Aerosolizes preformed nanoparticles	Reduces shear‐related variability	Preserves integrity and improves aerosol performance	Maintains encapsulation, including mRNA	Demonstrated in vivo functionality and safety	[46]

### 3.2. Particle Size, Shape, and Aerodynamic Considerations

Nanocarrier Physicochemical design plays a vital role in determining efficacy in pulmonary drug delivery since it directly affects particle deposition, retention, as well as curative results in the respiratory tract. The capability of inhaled formulations to make their way through the complicated architecture of pediatric airways is dictated by aerodynamics which are determined by particle size, shape, density, and surface characteristics [47, 48]. The ideal particles to use in deep lung deposition have a mass median aerodynamic diameter (MMAD) between 1 and 5 µm and particles larger than 5 µm settle in upper airway and those smaller than one settle in exhalation without effective deep deposition. Fine particle fraction, which is the proportion of inhalable particles that reach the alveolar regions, is a key measure in assessing the efficacy of formulations [49–51]. The fluidization, de‐aggregation, and deposition of the particle in the lower respiratory tract are also influenced by the morphology of the particle; high‐aspect‐ratio particles such as rods have better fluidization, deaggregation, and deposition than spheres. Such geometries also promote cell uptake and trafficking, like alveolar macrophages and epithelial cells, in targeted drug delivery [52, 53]. The likelihood of having an aerodynamic diameter is highest for particles of higher density and will be cleared from the lower respiratory system by mucociliary clearance, dependent upon the respective particle surface properties such as hydrophobicity, charge, and/or surface functionalisation. Examples of low‐density porous particle that include maintaining aerodynamic diameters that support deep lung penetration and reducing the phagocytic clearance, but surface‐engineered ligands are able to induce tissue uptake through receptor‐mediated endocytosis and targeted drug delivery [54, 55]. Furthermore, the additional factors for the pediatric population (reduced airway size, higher breathing frequency, formation of mucosal barriers, and others) further complicate these physicochemical parameters, and it is crucial to design the inhalable nanocarriers appropriately to guarantee optimal deposition and therapeutic efficiency. More recent research has demonstrated that it is possible to achieve a fine‐tuning of the particle size distribution, shape, and surface properties of the nanoparticles, for example, through the production of inhalable nanomedicines using spray‐drying and microfluidic‐assisted technology, which enables the generation of nanomedicines with predictable performance and scalable production capacity. Further, the integration of these design principles and controlled release formulations is useful in fulfilling a few of the unmet requirements in respiratory care of children [48, 56]. Conversely, if nanomedicine delivery is to be increased in children, then it is necessary to have more than just a dose reduction when compared to adult preparations to enhance the delivery of nanomedicines to the lungs. The respiratory system in children is different to that of adults in terms of airway size, lung volume, pattern of breathing, the nature of mucus, and use of devices, which plays a significant role in aerosol transport, deposition, and retention.

#### 3.2.1. Influence on Particle Deposition Mechanisms

The balance among inertial impaction, gravitational sedimentation and Brownian diffusion differs between children and adults because of anatomical and physiological differences. In infants, conducting‐to‐respiratory zone deposition ratios have been estimated to be 4.0 in infants, 0.5 in children, and 0.4 in adults, meaning that infants hold on to more micron‐sized particles in the conducting airways [34]. The total deposition level of 3 µm particles also increases with lower age subjects to reach ~88% in infants, 73% in children, and 66% in adults.

Inertial impaction and sedimentation are predominant with particles between 1 and 5 micrometers and smaller airway of pediatric lungs results in upper and conducting airway deposition [34, 57]. However, in comparison to nanoparticles that are smaller than 500 nm, Brownian diffusion plays a larger role. Simulation experiments of 5–500 nm particles in the airways of children revealed that deep airway deposition increased rapidly with the falling size of 3% at 500 nm to over 95% at 5 nm in distal small airways [32]. These data imply that the age‐based optimal particle size in pediatric delivery may depend on the target of therapy, disease condition, and delivery equipment as opposed to having an age‐independent universal range [31, 32, 34, 57].

#### 3.2.2. Pediatric‐Specific Mucus Properties

One of the significant factors in the transport and retention of nanoparticles is airway mucus. Pediatric patients with CF have hyperconcentrated mucus with increased levels of mucin and DNA. The CF‐induced bronchoalveolar lavage fluid contained almost 50% of nonsoluble mucus flakes instead of soluble mucus in preschool children [58]. There is also a significant increase in mucus dry weight, and this is linked to decreased mucociliary clearance. These characteristics form a denser and sticky shield that can inhibit the nanoparticles entry.

In various childhood respiratory conditions, mucociliary clearance is impaired. Longitudinal studies in children with CF have documented a median reduction in mucociliary clearance of 30% in children over 4.6 years, with *Pseudomonas aeruginosa* infection, a poorer lung clearance index, and increased structural lung damage showing poorer clearance [59]. The median mucociliary clearance at 60 min was 11.0–12.8% among early‐age children with CF [60]. Among children with allergic rhinitis, the nasal mucociliary transit time was greatly extended relative to controls with an average of 10.5 + 5.65 min versus 7.25 + 4.3 min [61], and a cutoff of over 535 s performed well as a diagnostic measure in one cohort of participants [62]. These findings used together suggest that a defect in the mucus clearance process could lengthen the residence time of nanoparticles in pediatric airways, which can enhance the local exposure but also pose a risk of toxicity.

There is limited direct pediatric evidence on mucus viscosity, layer thickness, and mucin composition [63]. Nevertheless, research on pediatric mouth‐throat models has demonstrated that the mucus rheology influences the transport of aerosols. Non‐Newtonian mucus altered the minimum particle size that left the trachea by ~60.2% relative to that of Newtonian mucus, which demonstrated the significance of rheology to influence downstream deposition [64].

#### 3.2.3. Particle Shape and Mucus Penetration

The shape of the particles is increasingly being identified to be a significant determinant of their transport by airway mucus, and there is a dearth of evidence specific to pediatrics. Adult preclinical experiments indicate that rod‐shaped or filamentous particles do not act like spherical particles with regard to diffusion, entrapment, and cellular uptake. Nevertheless, this relationship can be altered by the presence of altered mucus composition and rheology observed in pediatric disease, especially in CF [58, 59]. The optimization of shape of inhaled nanomedicines in children is speculative because pediatric data are not available and needs to be confirmed in pediatric‐relevant models [63, 64].

#### 3.2.4. Device‐Related Challenges in Pediatric Aerosol Delivery

The performance of the device used is a significant determining factor of effective lung dose in children. Nebulizers are popular among neonates and infants since they require no coordination, yet lung delivery is rarely greater than 10% of the nominal dose [65]. Mesh nebulizers have a better performance, and they have been able to deliver about 14% of the inhaled dose to the lungs in nasal or noninvasive ventilation circuits, though the majority of the dose is lost prior to reaching the lungs [24, 66].

Metered‐dose inhalers (MDIs) with valve holding chambers are popular in children and young infants; however, the administration also largely depends on the design of the chamber, the breathing pattern, and the time of actuation [67]. Losses are further increased by masks and nasal interfaces since the poor fit, leakages, and high levels of extra‐thoracic deposition cause significant attenuation of lung delivery with some studies showing losses of up to 50%–190% of emitted dose [31, 66, 68]. Nevertheless, aerosol delivery during noninvasive ventilation is especially difficult in the case of preterm babies due to the small anatomy, small lung volumes, and inconsistent breathing [69]. Inhalers containing dry powder are usually not recommended in infants and very young children since they need inspiratory flows of more than ~30 L/min [24].

#### 3.2.5. Implications for Nanomedicine Design

These characteristics unique to pediatrics have some implications to nanomedicine design. First, the size of the particle must be optimized to promote pediatric deposition behavior, and smaller nanoparticles have the potential to improve the ability to deliver drugs to the distal lung but are more associated with a higher risk of systemic absorption [32]. Second, mucus is another significant obstacle, particularly in CF, justifying the application of surface changes that can penetrate mucus or be used with mucoactive agents [58, 59, 70]. Third, particle shape is a variable that has not been settled in pediatric systems and must be treated with caution until pediatric data are available [63, 64]. Fourth, formulations should be nebulizer and pMDI‐valved holding chamber systems compatible, which represent major delivery systems in children [24, 65–67]. Lastly, adult‐to‐child dose scaling is not to be used in the pediatric dose selection since smaller lungs might be exposed to lower absolute dose but more local tissue [34].

### 3.3. Surface Chemistry, Functionalization, and Targeting Ligands

Surface charge, hydrophilicity, and chemical functionality can be modulated and allow the clearance and biodistribution mechanisms to be controlled precisely [71]. Endocytosis is aided by electrostatic adhesion of positively charged surfaces with negatively charged cellular membrane, and it can be detrimental because high cationic density can result in cytotoxicity and inflammatory responses; conversely, anionic and zwitterionic surfaces reduce nonspecific adsorption of proteins and prevent rapid clearance. Hydrophilic polymers polyethylene glycol (PEG) or poly(2‐oxazoline) polymer coating of nanoparticles provide steric stabilization and increased mucus penetration, but it needs to be optimized to avoid steric hindrance in viscoelastic airway mucus [72–74].

Bioactive ligands such as monoclonal antibodies, peptides, aptamers, or small‐molecule receptor agonists can be attached to the surface through chemical functionalization to selectively target alveolar epithelial cells, macrophages, or inflamed endothelium. Dual targeting strategies leverage off sequential receptor binding, for example, in circulating immune cells and at the tertiary site of lung epithelia, resulting in proinflammatory accumulation, while multivalent presentation of each ligand results in an improved caopicity of binding. Stimulus‐responsive surface functional groups, such as pH‐sensitive linkers, redox‐reactive disulfide bonds, or enzyme‐cleavable peptides, can be used to release payload controlled by pathological microenvironment and reduce off‐target toxicity. Surface topology and nanoscale roughness also affect opsonization, phagocytic recognition, and intracellular trafficking to provide further controls to delay and tune retention and release kinetics in alveolar space. Recent preclinical evidence indicates that the optimization of endosomal escape, transcytosis across the epithelial barrier, and intracellular bioavailability of encapsulated therapeutics can be improved upon with the engineering of nanoparticles with specific surface chemistry, which highlights the potential of ligand‐functionalized, surface‐engineered nanocarriers in lung disease treatment in children [75–77].

#### 3.3.1. Pediatric Evidence Synthesis

The available data remains very sparse as regards inhaled nanomedicine in the pediatric age, but several mechanistic lessons can provide valuable information to inform the design of formulations. The most informative themes in the existing data relate to the influence of the surfactant and stabilizer systems on the fate of nanoparticles, PEGylation effects on the penetration of mucus and aerosol stability, the influence of the formulation anchors on the transportation of epithelial cells, and the need to take into account age‐related problems of immune and safety [78, 79].

There is experimental neonatal evidence that the pulmonary fate of inhaled nanoparticles is extremely sensitive to the ambient surfactant climate and excipient selection. Surfactant‐stabilizer interactions showed a significant difference in nanoparticle half‐life and initial serum accumulation in the neonatal rat model where the stabilizer (Pluronic F127 or Poloxamer 188) was selected, demonstrating that surfactant‐stabilizer approaches can modulate retention, clearance, and systemic exposures upon pulmonary delivery. The results are of particular interest to pediatric nanomedicine since the surfactant environment in young life is not equal to that of adult lungs, and the choice of excipients is not only an active variable of the PK behavior but also a formulation variable [79, 80].

PEGylation has proven to be a very critical approach to enhancement of the performance of inhaled nanocarriers. Recent research on lipid nanoparticles (LNPs) and muco‐inert particulate systems as a whole indicates that a higher percentage of PEG correlates with an increase in resistance to nebulization‐induced shear stress, as well as an increase in diffusion despite mucus barriers [79, 81]. This is most applicable to respiratory delivery in pediatrics in which the mucus may be thick, sticky, and resistant to entry particularly during disease conditions. Nevertheless, the advantage of PEGylation seems not to solely rely on the density of PEG but also on the inclusion of PEG on the surface of the particles. In this regard, the brush PEG polymer‐based studies have revealed that the nature of lipid anchor can also play a significant role in mucus permeability, epithelial transport, and cellular uptake in rat models [82]. The implications of these results are that the balance between mucoadhesion and mucus penetration can be tuned by hydrophobic anchor selection, which is a primary design factor when targeting of the region in the lung is required in a way that prevents excessive trapping of particles in the airway secretions [78].

Juvenile safety‐relevant evidence, albeit limited, is direct and offers some positive evidence toward hesitant pediatric translation of PEGylated inhaled therapeutics. Repeat studies in juvenile rats using inhalation indicated that pegylated interferon *α*‐2b was well tolerated at relatively high doses and produced specific toxicokinetic profiles [83]. Although the results of such studies do not exclude the necessity of specific safety assessment of pediatric inhalers, the results are a valuable demonstration of a concept that PEGylated inhaled formulations may be systematically studied in immature organisms and may possess an acceptable safety margin under a controlled environment [82, 84].

The concept of immune‐targeting strategies is still appealing but has evidence mostly of the indirect type. Articles on dendritic cell‐targeted nanomedicines and inhaled nanoemulsion systems provide an indication of how to alter immune barriers in airways or induce immune tolerance [80, 85]. Nevertheless, the information on immune‐cell targeting, age‐specific inflammatory reactions, and long‐term immunological outcomes in direct pediatric data is extremely scarce. Currently, the majority of immunological assumptions used in pediatric inhaled nanomedicine are either extrapolated or simplified in vitro, which may not sufficiently capture the emerging immune environment of the developing lung in children [81].

Although these actionable insights exist, there are still critical evidence gaps. The cited studies lack direct human pediatric data on mucus composition, age‐stratified surfactant composition, and properties of airway lining fluids. The majority of the existing pediatric‐related experimental evidence is based on neonatal or juvenile rodent models [83, 86], which are a useful tool in mechanistic interpretation but cannot be completely translated into human pediatric airway anatomy, biochemistry, and immunology. More pediatric‐specific research is thus required to establish the interactions of surfactant composition, mucus architecture, epithelial development, and immune maturation that affect nanocarrier behavior following inhalation [85, 87].

### 3.4. Nanocarrier Classification Based on Composition

#### 3.4.1. Lipid‐Based Nanoparticles

LNPs can be used to deliver therapeutic agents such as oligonucleotides, mRNA, and CRISPR‐Cas9 as lipid‐based carriers to the lungs. They help guard against the destruction of nucleic acids and facilitate cell‐specific targeting such as lung macrophages do. Surveys have revealed the successful delivery of miR‐146a to reduce lung injuries and inflammation, and optimized LNPs enhance the delivery of mRNA and genome editing. They are versatile and hold potential in treating different respiratory diseases [88, 89]. Examples of different nanocarriers studied for pulmonary conditions are given in Table 2.

**Table 2:** Different types of nanoparticles for pulmonary conditions.

Type of nanoparticle	Target biomolecule	Conditions	Findings	Reference
Liposomes	rSLPI (recombinant secretory leukoprotease inhibitor)	Pulmonary inflammation	Liposome encapsulation improved rSLPI stability and reduced dosing frequency for inhaled therapy	[90]
Mannose‐decorated polymeric micelles	Rifampicin and curcumin	Pulmonary Tuberculosis	Mannose‐modified micelles enhanced macrophage targeting; 5.2× higher antimicrobial efficacy vs. unmodified micelles	[91]
Nanocomposites (nano‐in‐microparticles)	Rifampicin	Pulmonary Tuberculosis	Spray‐dried carbohydrate nanocomposites (3–6 µm); 89%–99% loading; respirable fraction 49.91%; reduced lung toxicity	[92, 93]
Nanoaggregates/trojan particles	Salmeterol xinafoate and Fluticasone propionate	Asthma	Polyamide‐based nanoaggregates (226.7 nm); 88%–98% release; superior aerodynamic performance vs. seretide diskus	[94]
Nanoaggregates (L‐arginine polymer‐based)	Thymoquinone + doxorubicin	Lung cancer	19.98 nm particles; high entrapement efficiency (>85%); effective deep lung delivery; strong therapeutic potential	[95]
Dendrimers (PAMAM G3)	TNF‐specific siRNA	Acute lung inflammation	Dendriplexes improved siRNA condensation and uptake; suppressed TNF in vitro and in vivo; effective anti‐inflammatory response	[96]

##### 3.4.1.1. Liposomes

Liposomes are spherical vesicles that have lipid bilayers that can entrap both hydrophilic and hydrophobic drugs. They provide chemical stability to the drug and can help in developing inhalable formulations for drugs needing deeper lung delivery. Folate‐modified remdesivir‐loaded functionalized liposomes have more targeting efficiency, long release, and therapeutic efficiency in viral infections and can also increase anticancer targeting. They are biocompatible and can be released on demand, for which reason they are utilized in providing medicines into the lungs [97, 98].

##### 3.4.1.2. Solid Lipid Nanoparticles (SLNs)

SLNs are capsules of hydrophilic and hydrophobic drugs that provide stability and biocompatibility. They increase pulmonary drug delivery by increasing lung deposition, avoiding first‐pass metabolism, and protracting drug release. Research on rapamycin‐ and carvacrol‐loaded SLNs demonstrates enhanced drug delivery, treatment efficacy, and decreased systemic lung damage because of the combination of solid and liquid lipids and enable high drug loading and regulated release [99, 100].

##### 3.4.1.3. Nanostructured Lipid Carriers (NLCs)

NLCs are second‐generation lipid NPs based on solid and liquid lipids that allow minimal lung damage, enhanced drug delivery, and therapeutic efficacy. Their size (less than 500 nm) helps increase lung deposition and retention and decreases the macrophage clearance. Their uses are antibiotics and antifungals with better efficacy, safety, and target against patients with pulmonary infections and other lung diseases [101].

##### 3.4.1.4. Lipid‐Based Hybrid Systems

The hybrid lipid‐polymer nanoparticles are a blend of lipid and polymer, which enhance the stability of the drug, its biovailability, and the ability to target the lungs. Surface‐active LPHNPs increase cell uptake, retention, and pulmonary accumulation. Research using voriconazole‐loaded LPHNPs demonstrate effective antifungal activity, enhanced lung residence, and improved therapy with minimal side effects [101, 102].

##### 3.4.1.5. Nano Emulsion

Nanoemulsions are oil–water dispersions with droplet sizes ranging between 20 and 200 nm, stabilized by cosurfactants and surfactants, which provide high bioavailability and increased lung absorption. DNA vaccines are delivered to the epithelial cells of the lungs and provoke an immune response using cationic nanoemulsions. Formulations of docetaxel, curcumin, and quercetin were optimized to show a good aerosol performance, deep lung deposition, and long‐term release of the drug. Nanoemulsions have the ability to deliver both hydrophilic and lipophilic drugs effectively, which aids in anticancer and anti‐inflammatory therapy [103].

#### 3.4.2. Polymeric Nanocarriers

##### 3.4.2.1. Nanospheres

Nanospheres are solid polymeric spheres that contain drugs in their center or on the surface. They are designed to be pulmonary to control release, increase lung retention, and increase absorption with minimal dosage and side effects. The ideal inhalable nanospheres are less than 200 nm, and their surfaces are high, biocompatible, and dispersible. Nanospheres composed of beclomethasone developed through evaporation of solvents enhanced the bacterial solubility, stability, and nebulization efficiency, which proved to be safe and effective in lung delivery [94].

**Figure 2:**
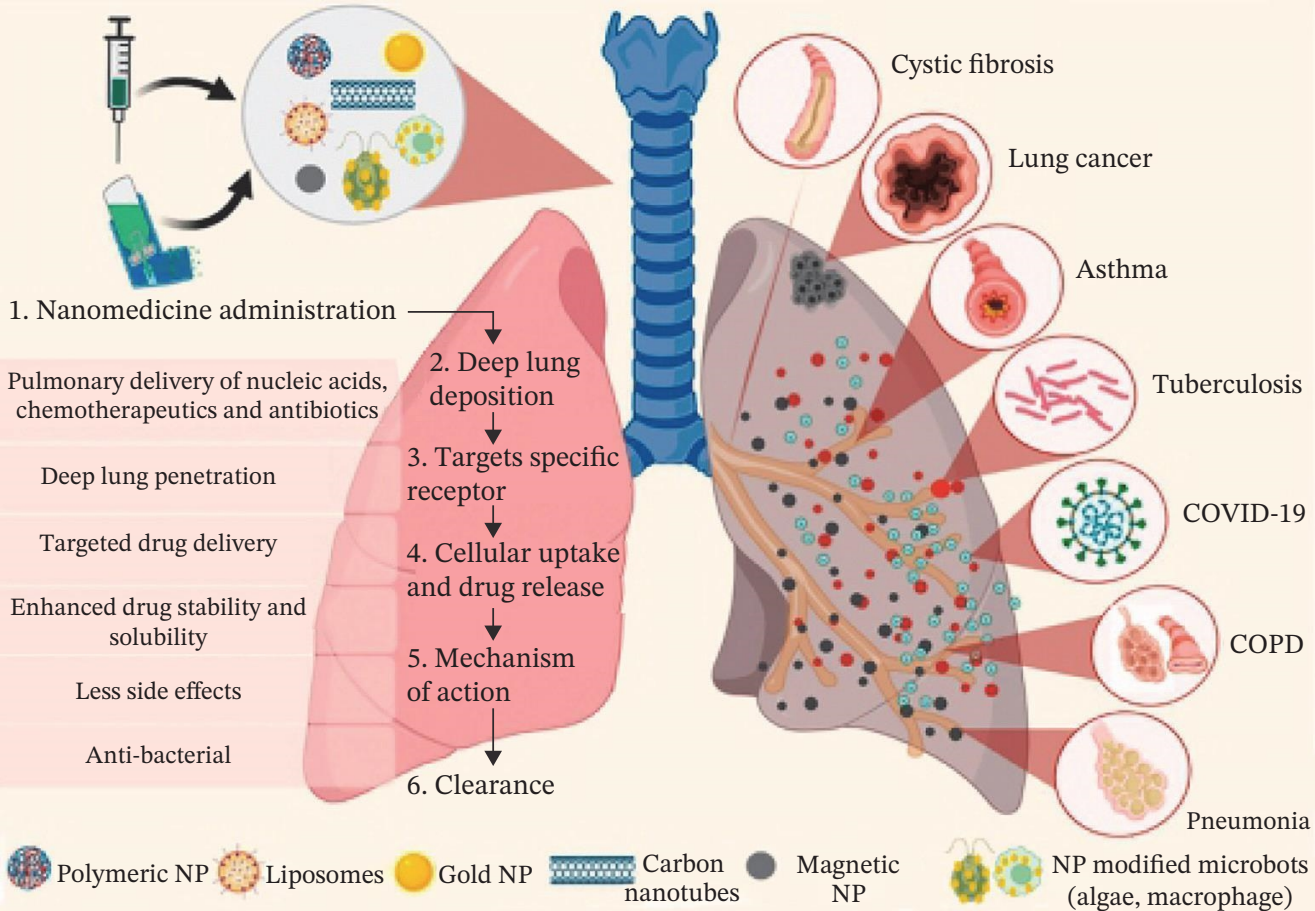
Schematic illustration summarizing the therapeutic landscape of nanomedicine‐based strategies for lung diseases. Adapted from Ref. [105].

Dendrimers are branched monodisperse macromolecules that can be loaded with drugs in the core or on the surface by covalent attachment. They facilitate targeted delivery of drugs, imaging, and diagnostics because of their ability to deliver drugs or probes such as radionuclides, fluorescent dyes, and MRI contrast agents. Dendrimers are suitable carriers to pulmonary use by their defined structure and versatility [104]. An outline of the therapeutic landscape of nanomedicine‐based approaches to lung diseases is shown in Figure 2.

##### 3.4.2.2. Polymeric Nanomicelles

Polymeric micelles (10–100 nm) are made of hydrophobic core and hydrophilic shell, which enhances the solubility of poorly water soluble drugs and allows a sustained pulmonary release. They decrease macrophage clearance, permit lung local delivery, and may penetrate mucus coats. Mixed micelles with paclitaxel loaded on lactose carriers were found to release drugs slowly, depose into the lungs effectively, and increase the cytotoxicity of paclitaxel on cancer cells. Mannose‐armed micelles that encapsulated rifampicin and curcumin showed high lung retention, enhanced microbicidal efficacy, and safe administration to the lungs. In inhalation therapy for cancer and tuberculosis, nanomicelles are used [106].

#### 3.4.3. Hybrid and Composite Nanocarriers

Nanocomposites, also known as nano‐in‐microparticles, are a mix of nanoparticles and microparticles that boosts lung deposition. The sugar matrices (150 sugar 100) containing drug‐loaded nanoparticles are dissolved in pulmonary fluids, and this releases the nanoparticles. Preparation is normally carried out using spray drying and spray‐freeze drying. This is because they avoid mucociliary clearance and macrophage uptake, which allows effective epithelial absorption. Rifampicin‐loaded nanocomposites exhibited excellent drug loading, enhanced inhalation efficacy, and less lung toxicity in pulmonary therapy [92].

#### 3.4.4. Nanoaggregates

##### 3.4.4.1. Specialized Formulation Approaches for Pulmonary Delivery

Nanosuspensions are colloidal suspensions formed by nanosized drug particles, which are stabilized using either polymers or surfactants. Methods such as spray drying using gel‐forming polymers produce stable, redispersible nanoagglomerates to be conveniently delivered into the air. Investigations using fluticasone propionate demonstrate that it has a longer lung retention, dissolution, and anti‐inflammatory properties. Tweens, Poloxamers and PVP are stabilizers that are used to stabilize the size of the particles. One highly beneficial use of nanosuspensions is in the pulmonary delivery of poorly soluble drugs [107]. Furthermore, nanoaggregates are hollow and merged microparticle and nanoparticle drug delivery. The spray drying is used to form viscoelastic shell which self‐assembles to nanoparticles to penetrate the deep lung, deliver uniformly, and be retained better. Research involving salmeterol xinafoate, fluticasone propionate, and thymoquinone shows high aerodynamic efficacy, precise release, and selective delivery, which makes nanoaggregates an apt choice in pulmonary delivery in respiratory and COVID‐19 treatment [108].

## 4. Nanomedicine‐Based Diagnosis of Pediatric Respiratory Diseases

### 4.1. Nanosensors for Pediatric Respiratory Diagnostics

**Figure 3:**
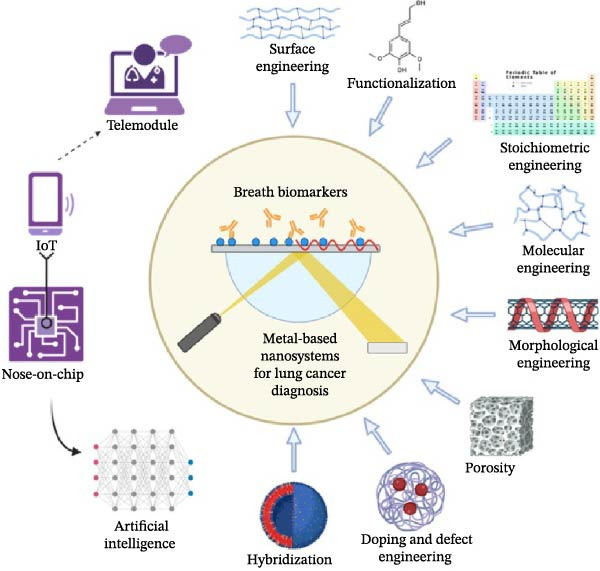
Schematic representation of engineered metal‐based nanosystems optimized via functionalization, doping, and hybridization strategies to enhance physicochemical performance for smart, point‐of‐care detection of lung cancer breath biomarkers. Adapted from Ref. [109].

Nanosensors represent a cutting‐edge diagnostic technology for noninvasive detection of respiratory diseases in children, allowing not only the identification of disease onset but also assessment of severity and progression, as summarized in Figure 3. These highly sensitive devices can detect specific biomarkers, including proteins, microRNAs (miRNAs), and other molecular indicators of disease. Plasma‐ and saliva‐derived exosomal miRNA profiling in pediatric asthma has been utilized to predict disease severity and responsiveness to therapy. Nanosensors have also shown promise in the rapid detection of pediatric pneumonia, where artificial biomarkers selectively interact with disease‐specific proteins to enable fast and accurate diagnosis. Moreover, nitric oxide (NO) and ozone, which are volatile and nonvolatile biomarkers, can be detected with nanosensors through the use of exhaled breaths to show that environmental triggers cause asthma attacks in children. Nanosensors in wearables are also being developed to monitor respiratory health on a continuous, real‐time basis to give clinicians information to change treatment on the spot. Despite their potential, challenges remain for clinical translation, including improving sensitivity, specificity, biocompatibility, cost‐effectiveness, and overall safety in pediatric populations, highlighting the need for further research to optimize these technologies for routine clinical use [110–112].

Biosensors based on nanoparticles hold promise as diagnostic agents to childhood respiratory conditions due to their ability to provide fast detection, high sensitivity, and have the potential to be used in point of care. Localized surface plasmon resonance sensors, magnetic nanoparticle‐based assays, as well as the gold nanoparticle lateral flow system or chemiresistive nanobiosensors can be used to reduce turnaround time and aid in the decentralization of testing during acute respiratory infections. Nevertheless, there is a dearth of clinical validation of the system in pediatrics, and most of them are in their initial phases of translation.

#### 4.1.1. Influenza Nanosensors

The concept of influenza nanosensors will help demonstrate the importance of quick multiplex testing in pediatrics. The RT‐LAMP assay with a lateral flow readout on a gold magnetic nanoparticle assay allowed the simultaneous detection of influenza A, influenza B, and an internal control, RNase P. The assay produced results in 35 min, had a limit of detection of 80 copies/mL, and 100% concordance with fluorescent PCR in a comparison of 70 samples. These types of prompt internally determined assays are applicable in case of respiratory outbreaks in children, where prompt diagnosis may enhance the use of triage and treatment decisions [113].

#### 4.1.2. RSV Nanosensors

The RSV is a significant pathogen among young children and infants, and thus quick diagnosis is of clinical significance. The spectral shift‐based localized surface plasmon resonance biosensors with metallic nanoparticles functionalized with anti‐RSV antibodies are used to sense viral binding. Compared to silver or gold nanoparticles, functionalized copper nanoparticles had superior limit of detection and limit of quantification, and both copper‐ and silver‐based systems were selective against adenovirus and *Pseudomonas aeruginosa*. These characteristics justify the prospect of the use of LSPR nanosensors as fast and relatively inexpensive substitutes to traditional RSV testing in the context of pediatrics [114].

#### 4.1.3. COVID‐19 Nanosensors

Nano‐diagnostic systems created to diagnose COVID‐19 demonstrate the overall ability of nanosensors to diagnose respiratory diseases. Nanoprobe‐based colorimetric sensors and magnetic nanoparticle‐enhanced immunoassays were developed to scale‐up signal, shorten the duration of the assays, and facilitate on‐site detection of SARS‐CoV‐2 antigens with ease. Such systems have pragmatic benefits like speedy color building, magnetic division, and easier procedures. Nevertheless, their validity is not always pediatric‐specific, and thus their applicability to child‐based respiratory diagnostics is only supportive as opposed to conclusive [115, 116].

#### 4.1.4. Tuberculosis Nanosensors

The use of nanosensors platforms can also increase the detection of TB in pediatrics, especially in under‐resourced regions where performing regular testing and sampling is difficult. To detect lipoarabinomannan, a chemiresistive biosensor was developed that is a paper‐based disposable sensor based on silver nanoparticles and MWCNT‐ZnO nanofibers. There was no given turnaround or numerical limit of detection as identified in the abstract, but the platform format was a fast, mobile, and low‐cost point‐of‐care format. The systems can be particularly applied to the setting of pediatric tuberculosis, whereby the diagnosis could contribute earlier to the detection of the cases and initiate the treatment [117, 118].

### 4.2. Imaging Modalities

Nanoparticle‐based imaging techniques, such as quantum dots (QDs), magnetic nanoparticles (MNPs), and gold nanosensors, can provide high diagnostic sensitivity, specificity, and real‐time imaging of diseased tissues in children with pulmonary diseases due to their high sensitivity and specificity. QDs are size‐tunable semiconductor nanocrystals with size‐dependent fluorescence that enable multiplexed cellular and molecular biomarker imaging of both lung tissues in addressing pediatric asthma (inflammatory proteins) and early‐stage lung fibrosis (morphology). Magnificent nanoparticles (especially iron oxide‐containing MNPs) are used as contrast agents in magnetic resonance imaging (MRI), improving the fire of structural abnormalities, airway inflammation, and infection in the lungs of children, and can give them useful data on tissue perfusion. Gold nanosensors leverage surface plasmon resonance properties to detect and image specific pulmonary biomarkers with high resolution, enabling real‐time monitoring of disease progression in CF and pediatric pneumonia [119, 120].

Moreover, the potential role of nanoparticles The imaging capabilities of pediatric respiratory nanomedicine are broadened with nanoparticles because of better lesion detection, inflammatory mapping, and cellular imaging with potentially lower doses. An iron oxide nanoparticle is used to create MRI nanoparticles, gadonium‐based nanoparticles are used to improve lesions, and inhaled iodinated nanoclusters are used to create CT nanoparticles. These systems can be applied to pediatrics since they are capable of supporting local imaging and dose reduction despite the majority of evidence being preclinical.

#### 4.2.1. Nanoparticle‐Based Imaging Agents

Nanoparticles made of iron oxide allow sensitive pulmonary inflammation and immune cell activity imaging. MRI signals of inflamed areas in lung disease models have been observed using antibody‐conjugated SPIONs to target subpopulations of alveolar macrophages. Experimental studies were performed on glycine‐coated SPIONs (~12 ± 5 nm core; ~84 nm hydrodynamic diameter) visualized in the lung and taken up by macrophages and neutrophils with no airway dysfunction or release of inflammatory cytokines. These results indicate that iron oxide systems can be used to do imaging of the cellular system with tolerable pulmonary tolerance in preclinical models [121, 122].

Structural imaging is improved by other nanoparticle systems. Gadolinium‐based nanoparticles when administered intratracheally were found to result in a high MRI signal sequence in fibrotic lesions (~120%) relative to healthy tissue (~50%), enhancing lesion imaging. Aerosol administration of inhaled iodinated nanoclusters elevated CT contrast by ~120 Hounsfield units in studies of animals, with no histologic lung damage [122, 123]. There is potential of dose‐sparing with targeted iron oxide particles, allowing high‐contrast imaging at 0.35 mg/kg (0.15 µg iron) dosages (0.35 mg/kg is equivalent to 0.15 µg/kg). It has also been examined in the context of dual modes with CT and MRI contrast agents [124].

#### 4.2.2. Pediatric Relevance and Safety Considerations

In the case of the pediatric use, dose reduction, local delivery, and pulmonary safety are important factors to be considered. The targeted iron oxide imaging at microgram‐level doses supports low‐dose feasibility [125], and inhaled nanoclusters have the potential to improve imaging with minimal systemic exposure [123]. The surface modification is also highly important since PEGylation and similar coating decrease oxidative stress and genotoxicity in relation to particles that are not coated. In preclinical studies, some preparations including glycine‐coated SPIONs have demonstrated good pulmonary tolerability with no airway dysfunction being detected [126].

Through all these progresses, pediatric clinical translation is still in the rest. The majority of the data are obtained using animal models, and others report transient oxidative stress or systemic distribution following an exposure to nanoparticles. Thus, as much as the nanoparticles imaging enhances the diagnostic aspect of the respiratory nanomedicine, there is need to optimize the dosage carefully, surface engineer, and special safety testing before the clinical practice can be adopted among the children [127].

### 4.3. Nanoparticle Hybrid Diagnostic Platforms

The nanoparticles used as signal amplifiers or capture probes of a certain biomarker (like cytokines, miRNAs, and pathogen‐associated antigens) in blood, saliva, and exhaled breath include gold and magnetic or polymeric particles. Microfluidic channels also provide control of biological fluids with high precision and therefore ensure effective interactions between nanoparticles and target molecules without requiring large volumes of the samples or long assays. With the utilization of MNPs in a microfluidic chip, the hybrid platform enabled the rapid detection of RSV in children with a results time of 30 min with a high sensitivity and specificity. Parallel measurement of several disease biomarkers is especially valuable in the diagnosis of complex diseases such as asthma or CF in children. A combination of nanotechnology and microfluidics offers these hybrid platforms, which could revolutionize pediatric pulmonary care by offering minimally invasive, real‐time, and highly accurate diagnostics [118, 128, 129].

### 4.4. Limitations of Pediatric Nanodiagnostics

Although respiratory nanodiagnostics is becoming increasingly popular, there is little translation in pediatrics due to a lack of clinical evidence in children, unfinished safety data, and big translation challenges. The majority of literature reviews focus on technical feasibility of preclinical promise, not strong pediatric validation, and thus, it is not clear how valuable these platforms really are in children. The current body of pediatric biomarker research is usually small, heterogeneous, and methodologically inconsistent, which restricts reproducibility as well as undermining clinical generalizability. Moreover, no standardized list of validated nanodiagnostic biomarkers or standardized protocols to address sample handling in common respiratory diseases have been found, which adds to the risk of false‐positive and false‐negative outcomes [130, 131].

The second important limitation is that the use of adult models or nonpediatric preclinical systems should be further maintained. Airway geometry, immune development, PKs, and clearance of nanoparticles cannot be directly extrapolated between children and adults. There is also no consideration of adult‐derived imaging endpoints and tolerability levels that show any important pediatric considerations, including reducing the level of sedation, radiation dose, device load, and sample size [132]. This leads to diagnostic performance estimated in adult or generalized models that can be a misrepresentation of efficacy as well as safety in infants and young children [131, 133].

Toxicology and device adaptation are to be determined. The nanodiagnostic systems used in pediatrics have to be small, of the right age, and suitable to low volumes of specimens and child‐centric processes. Nevertheless, practical issues of dosing control, safety of repeated use, and avoiding aggregation of particles or excessive exposures are not clearly covered [132, 133].

The role of the toxicological uncertainty is important, as well, since nanoparticles inhaled or delivered systemically can behave differently in the developing lungs and underdeveloped immune systems, and long‐term accumulation and cumulative effects in children are poorly understood. Further operational restrictions like the need to avoid sedation and the need to make caregivers acceptable further complicate clinical usage [133].

Translation is also hampered by reproducibility, production, and regulatory aspects. The formulation and variability in the production of nanoparticles reduce the interstudy consistency and make it challenging to conduct multicenter validation. Methods that perform well in controlled research setting are not necessarily scalable; they might not offer the quality control and standardization needed in the daily clinical practice. At the same time, the regulatory requirements in the pediatrics sector have not been established yet, and safety and efficacy are age‐related factors, which are usually difficult to address. As long as these issues remain unaddressed, it is likely that the pediatric respiratory nanodiagnostics will remain an appealing and underexplored technology that is not usable in a large‐scale clinical setting [134, 135].

## 5. Preclinical Evaluation and Animal Models

It is important to choose the right preclinical animal models that replicate the pediatric pulmonary anatomy and physiology in order to support the research on the inhaled nanomedicine. Inhalation‐based nanoformulations still rely on the use of rodent models (mice and rats) because of their ease of handling, cost‐effectiveness, and the availability of readouts on lung deposition, clearance, and pharmacodynamics, but their nasal breathing behavior and highly turbinated nasal cavities are markedly different from those of juvenile humans, and this affects aerosol deposition patterns and pharmacokinetics. Larger animal models (rabbits, guinea pigs, and dogs) provide larger tidal volumes and bronchial branching more reminiscent of those of humans, but with monopodial lung architecture as opposed to the approximately dichotomous branching pattern of human lungs, limiting complete anatomical congruence [136]. In studies relevant to neonatology or pediatrics, the study of fetal lung nanoparticle delivery in mice has provided valuable insights: one study revealed that intravenous injection of around 250 m PACE particles in fetal mice had appeared to deliver the particle to epithelial lung cells at a rate of ~50%, showing that pulmonary delivery is possible in immature lungs [137]. Furthermore, rodent ex vivo isolated perfused lung (IPL) models preserve integrity of alveolar/vascular interface and allow aerosol or instillation of nanoparticle formulations into perfused lung environment in a controlled setting allowing mechanistic analysis of deposition, clearance, and transport [138]. To translate pediatric inhalation nanomedicines disparity in airway diameter, lung compliance and the kinetics of mucociliary clearance and macrophage populations of young humans versus animal models need to be considered. Before clinical translation, a rigorous model selection that corresponds to pediatric lung morphometry is necessary to validate preclinical efficacy and safety data [137].

In studies of inhaled nanotherapeutics, it was revealed that formulations delivered by pulmonary route could attain lung‐to‐plasma AUC (area under the concentration–time curve) improvements of more than 10‐fold compared to systemic or oral delivery, and more than 40% of the intratracheally delivered dose was still in pulmonary tissue at 72 h postdose [139]. In a single study of the use of PLGA nanoparticles (Np, about 300 nm) as a drug carrier of the antifungal drug voriconazole, radiolabeling and gamma‐imaging of the particles showed that the NP accumulated in the lungs and cleared more slowly than the unencased drug: nanoparticles stayed in the pulmonary lobes, whereas the free drug was quickly cleared [140]. Also, gold nanoparticles of various sizes (2, 40, 100 nm) placed in the lungs of mice displayed a size effect translocation: the smallest (2 nm) was detectably translocated to liver, and the larger (~40–100 nm) was selectively accumulated in pulmonary macrophages and epithelial cells with little imaging in the [141]. These studies highlight that key determinants of PK/BD in pulmonary nanomedicines include particle size, surface charge/chemistry, aerosol aerodynamic diameter, physicochemical stability, and clearance pathway (mucociliary vs. alveolar macrophage). In practical terms, metrics such as lung tissue *C*_max_, *T*_1_/_2_ for lung retention, fraction of dose remaining in lung at defined timepoints (%ID/g), and off‐target organ distribution (liver, spleen, and kidneys) must be reported and interpreted in context of inhalation device, species model (rodent vs. larger mammal), and disease state. For pediatric translation, this is especially relevant, as children’s airway branching, lung volume, clearance kinetics, and macrophage populations differ markedly from adults. Thus, interpreting PK/BD data from preclinical models requires scaling and adaptation for the pediatric lung environment [142].

## 6. Clinical Translation and Pediatric Trials

Although the preclinical pipeline for inhalable nanomedicines in pulmonary disease is robust, actual clinical translation remains limited (Table 3). The Amikacin Liposome Inhalation Suspension (trade name Arikayce) represents a landmark milestone as the only FDA‐approved inhalable nanomedicine as of 2024, indicated for refractory pulmonary *Mycobacterium avium* complex (MAC) infections [142]. Other inhalable nanomedicine trials include lipid‐based mRNA delivery product LUNAR‐CFTR (ARCT‐032) targeting CF via aerosolised LNPs, now in Phase II (2025) following favorable early safety data [144]. In the realm of inflammatory and obstructive lung disease, the inhaled anticalin protein formulation Elarekibep (PRS‐060/AZD1402) (an IL‐4Rα antagonist) has progressed through Phase I into Phase II for asthma, showcasing how nanoparticle or protein‐nano hybrid systems are being clinically evaluated [144]. Unfortunately, despite high volume of preclinical work, meta‐analysis notes that only small fraction of inhalable nanoparticle systems have reached human studies, and many have stalled due to formulation/device scale‐up issues, aerosol stability, and regulatory hurdles [145].

**Table 3:** Clinical development of inhalable nanomedicines for pulmonary infectious diseases.

Trade name	Nanoparticle type	Indication	Clinical trial status
Arikayce	Liposomal amikacin	Nontuberculous mycobacterial (NTM) lung infection caused by *Mycobacterium avium* complex (MAC)	FDA‐approved (2018); EMA‐approved (2020) [143]
Amikacin Liposome Inhalation Suspension (ALIS)	Liposomal amikacin	NTM lung infection due to MAC	Phase III (NCT04677569)
Arikace	Liposomal amikacin	Cystic fibrosis (CF) with *Pseudomonas aeruginosa* infection	Phase I/II (NCT00558844)
Arikayce	Liposomal amikacin	CF with *P. aeruginosa* infection	Phase I (NCT05999942)
Arikayce	Liposomal amikacin	Chronic *P. aeruginosa* infections in CF patients	Phase III (NCT01315678)
Arikayce	Liposomal amikacin	Bronchiectasis with chronic *P. aeruginosa* infection	Phase II (NCT00775138)
Arikayce	Liposomal amikacin	CF patients with *P. aeruginosa* infection	Phase I/II (NCT00777296)
Arikayce	Liposomal amikacin	CF with *P. aeruginosa* infection	Phase III (NCT01315691)
COVID‐19EXO	Exosome‐based formulation	COVID‐19–associated pneumonia	Phase I/II (NCT04491240)
COVID‐19EXO2	Exosome‐based formulation	COVID‐19–associated pneumonia	Phase II (NCT04602442)
Liposomal Amikacin for Inhalation (LAI)	Liposomal amikacin	NTM lung infection due to MAC	Phase III (NCT02344004)
Liposomal Amikacin for Inhalation (LAI)	Liposomal amikacin	*P. aeruginosa* infections in CF patients	Phase III (NCT01316276)
Liposomal Amikacin for Inhalation (LAI)	Liposomal amikacin	NTM lung disease caused by MAC	Phase III (NCT02628600)
Liposomal Amikacin for Inhalation (LAI)	Liposomal amikacin	*Mycobacterium abscessus* lung infection	Phase II (NCT03038178)
Liposomal Amikacin for Inhalation (LAI)	Liposomal amikacin	NTM infections	Phase II (NCT01315236)
Liposomal Amikacin for Inhalation (LAI)	Liposomal amikacin	CF patients with *P. aeruginosa* infection	Phase II (NCT03905642)
Liposomal Amikacin for Inhalation (LAI)	Liposomal amikacin	*Mycobacterium xenopi* lung disease	Phase II (NCT06585020)
Pulmaquin	Liposomal ciprofloxacin	Noncystic fibrosis bronchiectasis	Phase III (NCT01515007)
Pulmaquin	Liposomal ciprofloxacin	Non‐CF bronchiectasis	Phase III (NCT02104245)
Pulmaquin	Liposomal ciprofloxacin	Non‐CF bronchiectasis	Phase III (NCT01515007)
Ambineb	Liposomal amphotericin B	Pulmonary aspergillosis	Phase II (NCT00391014)
Liposomal Amphotericin B (ALN)	Liposomal amphotericin B	Pulmonary aspergillosis	Phase II (NCT04267497)
Liposomal Amphotericin B (ALN)	Liposomal amphotericin B	Pulmonary aspergillosis	Phase II/III (NCT00263315)
AmBisome	Liposomal amphotericin B	Pulmonary mucormycosis	Phase II (NCT04502381)
Abelcet	Liposomal amphotericin B	Prevention of pulmonary fungal infections	Phase III (NCT00177684)
AmBisome	Liposomal amphotericin B	Pulmonary aspergillosis	Phase III (NCT03656081)
AmBisome	Liposomal amphotericin B	Pulmonary aspergillosis	Phase II (NCT03327727)
Remdesivir (GS‐5734)	Nanocrystal remdesivir	COVID‐19 and SARS‐related pneumonia	Phase I (NCT04480333)

### 6.1. Age‐Dependent Deposition and Regional Distribution

Children are not just different to adults based on the size of the lung but also regional deposition behavior. In infants and young children, smaller airways, smaller lung volumes, and increased respiratory rate are associated with more proximal deposition, whereas in older children and adults, more alveolar deposition is attained [34, 146, 147]. Simulations of whole lungs revealed that total deposition of a 3 μm aerosol in infants was ~88% in comparison with about 66% in adults with a ratio of conducting to respiratory deposition being 4.0 in infants and 0.4 in adults. These data show that the pediatric lungs are likely to be exposed to a greater local airway burden despite less deep lung targeting. This relationship is further altered by breathing pattern and route of inhalation, with the smaller tidal volumes, the increased respiratory rate, and the age‐related nasal deposition potentially causing significant variation in the fraction reaching the lower airways [146].

### 6.2. Clearance and Systemic Availability

Centrally deposited material in children is eliminated by a rapid mucociliary mechanism, and the deeper deposits by a slower bronchial or alveolar mechanism. The modeling studies indicate that the velocity of the mucus can be reduced in young individuals, but because the path length of the mucocilia in small lungs is always shorter, it can continue to provide relative efficiency in central clearance. Conversely, slower clearance of the alveoli is seen to be less affected by age, and this may allow peripheral deposits to be retained longer. Inhalation Systemic exposure is a indicator of pulmonary absorption and gastrointestinal absorption of the swallowed fraction, and this contribution varies according to product and device. Systemic availability in preschool children receiving nebulized budesonide was ~6.1% of nominal dose, 26.3% of nominal dose given to subject, and inhalation clearance was 0.54 L/min with a half life of ~2.3 h [148]. The intravenous beclomethasone data in children aged between 10 and 14 years demonstrated a clearance of ~0.9 L/min and a terminal half‐life of ~1.7 h of the active metabolite [146, 147, 149].

### 6.3. Measured Lung Delivery and PK Bridging

Pediatric trials indicate significant differences in the delivered lung when using devices and formulations. Pharmacokinetically estimated mean lung deposition in children aged between 8 and 14 years was 30.8% of budesonide administered by Turbuhaler and only 8% by Diskus. The average dose to the subject of nebulized budesonide in children aged 3–6 years was ~23% of the nominal charge. In the case of hydrofluoroalkane beclomethasone in children, ~82% of the available drug in the body was absorbed via the lungs and 18% via the gastrointestinal tract [149–151]. These results indicate that pediatric PK analyses have the capability to suggest useful estimates of lung dose and systemic exposure that are more informative than nominal device dose on its own. Inhalation‐specific child profiles, mouth‐throat models, in vitro deposition testing, and PK confirmation have been used together to make predictions of the pediatric dose‐to‐lung of delivery systems, for example, Respimat [152].

### 6.4. Limitations of Simple Dose Scaling

Direct scaling of adult inhaled doses based on body weight, body surface area, or tidal volume does not work in children due to changes in deposition fraction, regional distribution, clearance, and systemic handling with age and equipment. This can be seen in the significant difference in the amount of pediatric lung deposited using inhaler systems such as 30.8% versus 8.0% measured in the children using two different corticosteroid products [149, 151]. Nebulized budesonide trials also indicate that down‐scaling at mg/kg can be inaccurate since systemic management is not always proportional to adults [148]. Age‐specific anatomy, breathing measurements, particle and device profiles, deposition patterns, and PK target studies of inhaled drug delivery should instead be integrated to optimize the inhaled dose in young people than a scaling rule based on adult studies [148–153].

### 6.5. PK Modeling and Dose Optimization

PK modeling especially the physiologically based pharmacokinetic (PBPK) models has, over recent years, become a vital method to optimize the dosage in children [154]. PBPK models permit a mechanistic insight into drug absorption, distribution, metabolism, and excretion (ADME) within the body. An example is a study that scaled up an adult azithromycin PBPK model to a pediatric case that factored age‐related organ maturation and physiological alterations [155]. Renal impairment (RI) is further complications of dose selection as Rahim et al. have created a PBPK model of meropenem to maximize dosing in children with different levels of RI [156, 157]. Salivation PK profiles were very similar to plasma data, and the evidence supporting saliva as a surrogate in pediatric research was validated [156]. There are also PBPK models used to optimize doses of various types of drugs [158].

Ethical aspects and stringent regulatory issues dominate the production of nanomedicines that can be inhaled by children because of physiological susceptibility in children. The regulatory bodies (the U.S. Food and Drug Administration [FDA], European Medicines Agency [EMA], and International Council for Harmonisation [ICH]) are used to guarantee that the development of children drugs is safe, effective, and scientifically warranted. EMA requires Pediatric Investigation Plan (PIP) to be submitted to all new drugs, including nanomedicines, to make sure that age‐specific formulations, dosing, and safety evaluations are taken into account. Pediatric Study Plans (PSPs) are required by FDA that describe the clinical trial design and endpoints and safety monitoring in relation to age. Such frameworks make sure that the ethics of pediatric research are justified and scientifically sound (European Medicines Agency, 2023). Pediatric clinical research heavily relies on ethical supervision. Trials should focus on the minimal invasiveness of inhalable nanomedicine, and noninvasive sampling techniques should be used. The principles of beneficence, non maleficence and consent of older children as well as parental consent are essential. The study protocols ought to be regulated under Institutional Review Boards (IRBs) or Ethics Committee as these protocols must take into consideration the potential risks and benefits that are expected to be obtained [159].

The biodistribution and immunogenic properties of nanoparticles may be different in children. The regulatory guidelines suggest that prior to first‐in‐child studies, a considerable amount of preclinical safety testing in age‐suited animal models should be conducted. The parameters that should be determined include pulmonary deposition, systemic absorption, immunotoxicity, and long‐term biocompatibility to avoid the adverse effects [160]. Age‐specific devices to inhalation therapy in children are necessary, such as nebulizers, dry‐powder inhalers (DPIs), and MDIs. Regulatory authorities demand showing that the dose delivery is reproducible, as well as the performance of the aerosol and the usability of the devices in the pediatric groups. Adjustments to younger children, like mask‐assisted delivery or the use of spacer devices, are usually required to assure efficient pulmonary deposition [161]. Nanomedicines in the pediatric setting should be carefully evaluated in terms of risks and benefits. Although approval is given, postmarketing surveillance (Phase IV) is required to observe long‐term safety, possible developmental impact, and unusual adverse events. In many cases, regulatory authorities demand the existence of registries and pharmacovigilance plans related to pediatric groups (FDA, 2022) [162].

## 7. Safety and Toxicology in Pediatric Nanomedicine

Children possess smaller airways, faster breathing rates, and an immature mucociliary clearance system, which may affect deposition, absorption, and clearance of nanoparticles in lungs. When creating, dosing, and evaluating inhalable nanotherapeutics, special consideration needs to be given to physiological differences [163]. There is immaturity of the alveoli surface area and branching, especially in neonates and infants. Therefore, inhaled particles of nanoparticles may exhibit altered deposition patterns that lead to nonuniform deposition or over deposition in various regions of the lung. It has been demonstrated through preclinical experiments in young animal models that nanoparticles below 100 nm can be absorbed deep into the alveolar space, which may enhance systemic absorption and off‐target exposure. Thus, optimization of the size of the particles is essential to obtain therapeutic pulmonary delivery and reduce systemic exposure [31]. Pediatric immune system is not fully developed and that may have an impact on efficacy and safety of inhalable nanomedicines in both therapy and safety. Nanoparticles have the ability to initiate innate immune responses, which include macrophage activation, cytokine release, and complement activation. The most frequent approach is the use of surface modification methods, like PEGylation or biocompatible polymer surface coating that aim to decrease immunogenicity and increase tolerability [164]. Repeated or chronic exposure to the nanoparticles increases bioaccumulation, especially in the case of nonbiodegradable or noncleared material. Pediatric patients are more susceptible since their developing organs might be more vulnerable to long‐term exposure and their lifetimes mean that they can accumulate. In juvenile rodent model studies, chronic nanoparticles were observed to accumulate in lungs, liver, and spleen, and this may impact organ development and functioning. Such risks can be alleviated by using biodegradable nanoparticles or by creating stimuli‐responsive carriers [165].

Normal doses based on body weight might lead to either overexhibition or under‐therapeutic effects in the adult form. PBPK modeling Physiologically based, PBPK modeling is becoming increasingly popular in predicting the systemic exposure and lung deposition in pediatrics, which can assist in optimizing the safe and effective dose [166]. Children with chronic lung diseases (asthma, CF, and bronchopulmonary disorders of the newborn) and altered airway geometry, mucus, and inflammatory condition contributing to nanoparticle toxicity, or changes in drug delivery efficiency, include premature infants and children with chronic lung disease. Nanoparticles coated with muco‐inert or mucus‐penetrating targets are in development to pass these barriers with minimum local irritation [167]. The rational design, surface engineering, and overall biocompatibility testing are the areas of mitigation of nanoparticle‐induced toxicity. Instead, biodegradable polymers like PLGA, chitosan, and lipids are used since they decompose into nontoxic metabolites, lowering the formation of ROS and inflammation. PEGylation or hydrophilic surface modifications are surface modifications that decrease immune recognition and lower the induction of cytokines. Multitier biocompatibility involves in vitro cytotoxicity and ROS tests, ex vivo lung tissue experiments, and juvenile animal models to determine lung retention, clearance, and immune response. NF‐KB and inflammasome activation inhibition via immunomodulatory approaches like incorporation of anti‐inflammatory coating or dexamethasone‐functionalized nanoparticles, which have shown safety in pediatric inhalation models, have been demonstrated. PK modeling‐based dose optimization yields therapeutic doses without surpassing toxicity limits [168, 169]. Additionally, NPs, because of their small size, high surface area, and unusual physicochemical characteristics, have the ability to react with cellular and molecular components in a nontraditional manner, unlike conventional drugs [170]. Main pathways where nanoparticles cause toxicity is by producing reactive oxygen species (ROS). Too much ROS may cause oxidative stress, which destroys lipids, proteins, and DNA. In inhaled NPs, the initial site of exposure is the lung epithelium, and oxidative stress may lead to damage of barrier integrity and induce apoptosis and inflammatory signaling. Metal oxide nanoparticles (ZnO and TiO_2_) and certain polymeric NPs have been demonstrated to elevate the ROS levels in the epithelial cells and macrophages of the alveoli, resulting in dysfunction of the lungs in preclinical research. Surface modifications and polymer coatings by employing antioxidants are commonly used to alleviate ROS‐mediated damage [171]. The nanoparticles may also induce inflammasome activation, which are cytosolic multiprotein complexes that control the maturation of proinflammatory cytokines (IL‐1 8). The NLRP3 inflammasome is specifically involved in the inflammatory process caused by nanoparticle in the lungs. The assembly of NLRP3 can be facilitated by NP uptake by alveolar macrophages, lysosomal damage, or ROS production, resulting in caspase‐1 activation and downstream inflammation. Chronic inflammasome activation plays a role in chronic lung inflammation, tissue remodeling and fibrosis in preclinical models. Toxicity associated with inflammasomes can be diminished via surface functionalization and biodegradable preparations [172, 173]. Another important mediator of NP‐induced pulmonary toxicity is the nuclear factor kappa‐light‐chain‐enhancer of activated B cells (NF‐*κ*B) signaling pathway. NF‐xB may be triggered by exposure to NPs, particularly those with cationic charge or high aspect ratio and lead to transcription of proinflammatory cytokines (TNF‐x, IL‐xx, and IL‐xx). NF‐*κ*B signaling Chronic NF‐*κ*B activation can intensify inflammatory lung diseases, such as asthma and chronic obstructive pulmonary disease (COPD), and is one of the reasons to consider when designing inhaled therapeutics to be used in children. NF‐cB‐mediated inflammation can be dampened by changing the particle charge, size, and surface chemistry [163, 170].

## 8. Future Perspectives

The rational design of nanocarriers to deliver drugs into the lungs is being transformed by artificial intelligence (AI) and machine learning (ML). Prediction of nanoparticle stability, drug loading efficiency, and biodistribution can be performed by AI algorithms on the basis of physicochemical properties and saves a large number of experimental iterations. Quantitative structureactivity relationship (QSAR) models also allow high‐throughput screening of nanomaterials, derivation of molecular characteristics of toxicity, immunogenicity, and therapeutic efficacy. These computational systems are able to determine the best particle size, surface charge, and hydrophilicity for improving lung deposition and cellular uptake with minimum ROS generation and provocation of immune responses and inflammatory reactions. AI‐assisted personalized inhalation treatments allow patient‐specific factors to be taken into account, such as the type of disease, lung volume, and age. Systemic exposure in pediatrics and prediction of drug release kinetics can be accomplished by integrating with PK modeling. Moreover, AI can help forecast long‐term safety outcomes, such as possible chronic pulmonary toxicity. In silico strategies can shorten the distance between nano medicine development as preclinical models and clinical trials, enhance efficiency in design, and lower costs [174–176].

Nanotheranostics combine diagnostic and therapeutic capabilities onto one nanoparticle platform and provide the first‐ever capability to monitor drug delivery and treatment response in real time. Therapeutic payloads can be coloaded with imaging agents like MRI contrast dyes, radionuclides, or fluorescent probes and therefore can be visualized and therapeutically treated at the same time. Preclinical research shows that theranostic dendrimers and liposomes have the potential to monitor inflammation on‐the‐fly and deliver anti‐inflammatory or antimicrobial drugs. This dual capability is specifically applicable to the pediatric demographic where noninvasive imaging with a corresponding localized therapy can minimize systemic toxicity and increase the level of treatment compliance. Stimuli‐responsive release mechanisms and multifunctional coating further improve the accuracy and safety of such platforms. Continued studies are underway on the application of AI on automated imaging feedback analysis, allowing adaptive therapy dosing [177–179]. Phosphorene, MXenes, and black phosphorus nanosheets are biodegradable 2D nanomaterials, which are emerging as the next generation of carriers of inhaled therapeutics. These ultrathin materials provide high surface area, tunable drug loading, and a rapid biodegradation process to nontoxic metabolites. Their planar geometry enables superior penetration of the mucosa and a longer retention at the alveolar regions, enhancing therapeutic effects. PEGylation, polymers, or targeting ligand functionalization can regulate immunogenicity and minimize toxicity caused by ROS. It has been demonstrated in preclinical research that biodegradable 2D nanocarriers have the capacity to codeliver several drugs or gene therapies and show synergistic effect in pulmonary infections and inflammatory lung illnesses. This is combined with smart inhalation devices to provide accurate dosing and deposition [180, 181].

For successful clinical translation of inhalable nanomedicines, harmonized regulatory frameworks to ensure the safety, efficacy, and reproducibility of nanomedicines need to be established. The current regulations are region‐specific to address aspects relevant to differences between regions, including the use of novel nanotherapeutics in pediatrics, which limits the global use of novel nanotherapeutics. Standardization of physicochemical properties of nanoparticles and toxicological and PK characterization are all included in regulatory harmonization. For regulatory approval, pediatric‐specific safety information, including long‐term pulmonary and immune follow‐up, is required. Advanced modeling, in vitro humanized lung models, and AI‐driven toxicity prediction can be employed to aid in regulatory submission and reduce the need for large animal use. Interdisciplinary working models that involve the research institutes, industry, and regulatory bodies, which also have a significant role in the improvement of the process of clinical trials translation need to be implemented. In order to close this gap, there is a need for unified regulatory approaches that ensure the safe and effective use of innovative nanomedicine products in children [182].

## 9. Conclusion

Inhalable nanomedicines are paradigm shifts to tackle pediatric and adult lung diseases, bridging mechanistic understanding, preclinical and clinical translation approaches. Mechanistic studies also highlight the ability of the various nanocarriers to make drugs more soluble, to uptake into cells, and to deliver targeted drug delivery without inducing systemic exposure and off‐target toxicity. Early research has highlighted the critical role of particle size and surface chemistry and biocompatibility in achieving optimal lung deposition, retention, and therapeutic potential in infectious and inflammatory lung diseases. In PK modeling and dose optimization in the pediatric population, the opportunity to individualize inhaled therapy, increase the level of safety and efficacy of the therapy, and take into account the specificities of the children’s anatomy and physiology. Inhaled nanotherapeutics have been proven to be both feasible and effective through a multitude of trials of liposomal amikacin, amphotericin B, or newer exosome‐based therapies, which have been clinically translated to date. Nevertheless, there are still difficulties in the alignment of regulatory systems, long‐term safety, and alleviation of immunological risks, especially among children. The new nanotechnologies, such as the use of AI to design nanocarriers, nanotheranostics, and 2D materials with stimuli‐responsive properties, would also further accelerate the translative process through precision and real‐time monitoring and combinatorial therapy. The integration of concepts will need to address the current bottlenecks to make inhaled nanomedicines a safe, effective, and patient‐specific therapeutic option. The development of computational modeling, preclinical validation, and regulatory thinking on integrating concepts will be a key enabler in achieving this goal.

## Author Contributions

All authors made a significant contribution to the work reported, whether that is in the conception, study design, execution, acquisition of data, analysis and interpretation, or in all these areas; took part in drafting, revising, or critically reviewing the article.

## Funding

The authors have nothing to report.

## Disclosure

All authors gave final approval of the version to be published, agreed on the journal to which the article has been submitted, and agree to be accountable for all aspects of the work. All authors listed meet the ICMJE criteria for authorship, have read the final manuscript, and agree to publish this work.

## Ethics Statement

This is a review paper and does not involve direct research on humans or animals.

## Consent

The consent is not applicable as this manuscript does not contain data from any person.

## Conflicts of Interest

The authors declare no conflicts of interest.

## Data Availability

Data sharing is not applicable as no new data were created or analyzed in this study.
